# Macrophage‐derived exosomal miR‐4532 promotes endothelial cells injury by targeting SP1 and NF‐κB P65 signalling activation

**DOI:** 10.1111/jcmm.17541

**Published:** 2022-09-07

**Authors:** Peng Liu, Shuya Wang, Guangxin Wang, Mingming Zhao, Fengli Du, Kaiyuan Li, Lei Wang, Huihui Wu, Jiamin Chen, Yang Yang, Guohai Su

**Affiliations:** ^1^ Central Hospital Affiliated to Shandong First Medical University Jinan Shandong China; ^2^ Research Center of Translational Medicine, Jinan Central Hospital Cheeloo College Shandong University Jinan Shandong China; ^3^ Shandong Provincial Public Health Clinical Center Jinan Shandong China

**Keywords:** atherosclerosis, endothelial cells, exosome, macrophages, miR‐4532, NF‐κB P65, SP1

## Abstract

Atherosclerosis is a complex pathological process involving macrophages, endothelial cells and vascular smooth muscle cells that can lead to ischemic heart disease; however, the mechanisms underlying cell‐to‐cell communication in atherosclerosis are poorly understood. In this study, we focused on the role of exosomal miRNAs in crosstalk between macrophages and endothelial cells and explored the rarely studied molecular mechanisms involved. Our in vitro result showed that macrophage‐derived exosomal miR‐4532 significantly disrupted human umbilical vein endothelial cells (HUVECs) function by targeting SP1 and downstream NF‐κB P65 activation. In turn, increased endothelin‐1 (ET‐1), intercellular cell adhesion molecule‐1 (ICAM‐1) and vascular cell adhesion molecule‐1 (VCAM‐1) and decreased endothelial nitric oxide synthase (eNOS) expression in HUVECs increased attraction of macrophages, exacerbating foam cell formation and transfer of exosomal miR‐4532 to HUVECs. MiR‐4532 overexpression significantly promoted endothelial injury and pretreatment with an inhibitor of miR‐4532 or GW4869 (exosome inhibitor) could reverse this injury. In conclusion, our data reveal that exosomes have a critical role in crosstalk between HUVECs and macrophages. Further, exosomal miR‐4532 transferred from macrophages to HUVECs and targeting specificity protein 1 (SP1) may be a novel therapeutic target in patients with atherosclerosis.

## 1 INTRODUCTION

Atherosclerosis is the main cause of coronary heart disease, cerebral infarction and peripheral vascular diseases.^1^, ^2^ Endothelial cells constitute physiological barriers, which sustain normal vascular function.^3^ High glucose, circulating low‐density lipoprotein or Apolipoprotein B, accumulate beneath the vascular endothelial cell layer and recruit macrophages to participate in a series of adverse, non‐resolving inflammatory reactions that accelerate endothelial inflammatory responses and promote atherosclerotic plaque formation.^4^ Recent research has verified that nitric oxide synthase 1‐derived nitric oxide and the small heat shock protein, HSP27, are involved in macrophage and endothelial cell interaction and related to apoptosis and autophagy.^5^, ^6^ During cell activation and apoptosis, macrophages can release several types of membrane‐bound extracellular vesicles, including exosomes, microvesicles/microparticles, and apolipoproteins, and whether or not these vesicles are involved in atherosclerosis remains unclear.^7^

Endogenous and highly conserved non‐coding RNAs have important roles in atherosclerosis pathophysiology by regulating atherosclerosis susceptibility genes and influencing post‐transcriptional gene regulation. Non‐coding RNAs control the expression of approximately 30% of genes at the post‐transcriptional level,^8^ and increasing numbers of studies have revealed the importance of miRNAs in regulating key signals and lipid homeostasis pathways that alter the balance of atherosclerotic plaque progression and regression.^9^ Increased circulating levels of miR‐208a, miR‐29 and miR‐155 have been reported in patients with slow coronary flow (SCF), and proposed as promising biomarkers for SCF diagnosis.^10^ Some researchers have also speculated that specific miRNAs may serve as biomarkers for rupture‐prone plaques,^11^ and circulating miR‐155 plasma levels are decreased in patients with unstable angina or myocardial infarction.^12^ Thus, levels of miRNAs circulating in blood exhibit great potential as predictive biomarkers of disease progression.^13^

Exosomes are nano‐sized small extracellular vesicles secreted by cells and which carry substances, such as nucleic acids, proteins and lipids, thereby influencing physiological and pathological processes. Exosomes contain numerous components of their cells of origin, including DNA, RNA and proteins, among other factors.^14^, ^15^ Non‐coding RNAs carried in exosomes play important roles in intercellular communication, particularly in atherosclerotic disease, where exosomes act as vehicles that tightly link macrophages, smooth muscle cells and endothelial cells.^16^, ^17^ There have been a relatively large number of studies of the interactions between macrophages and smooth muscle cells via exosomes; however, investigation of crosstalk between macrophages and endothelial cells via exosomes remains scarce. Macrophages and endothelial cells are interconnected and accelerate atherosclerosis development through exosomes.^18^, ^19^ Atherosclerosis development is initiated by entry of low‐density lipoprotein (LDL) cholesterol into the arterial wall and its phagocytosis by macrophages, leading to foam cell formation and atherosclerosis development. Macrophage‐produced exosomes contain anti‐inflammatory microRNA‐99a/146B/378a that stabilize atherosclerotic plaques,^20^ and endothelial cell miR‐92a can be transported to macrophages through extracellular vesicles to accelerate the development of atherosclerosis.^19^, ^21^ In addition to the most commonly studied cells, such as macrophages and smooth muscle cells, when dendritic cells, platelets and other cells are stimulated by ox‐LDL, they can all secrete miRNA encapsulating exosomes which modulate inflammatory responses and intercellular adhesion molecule expression in endothelial cells and have potential to regulate alterations in neutrophil reactive oxygen species levels and net release.^22^, ^23^, ^24^ All of these factors play important roles in atherosclerosis progression.

SP1 is involved in regulating many pathological processes, including cardiomyocytes apoptosis, oxidative stress, vascular endothelial cell injury and lipid deposition, which in turn affect the development and progression of diseases, such as coronary heart disease, cardiomyopathy and atherosclerosis.^25^, ^26^ NF‐ κB, as a proinflammatory factor, is an important inflammatory driver in atherosclerosis development.^27^ Regulation of NF‐ κB and SP1 expression may be important targets for the prevention and treatment of atherosclerotic vascular disease.^28^

Overall, reports of the underlying mechanisms associated with macrophage‐endothelial cell‐contact are rare, and more investigations to explore this area are needed. In this study, we aimed to identify exosomal miRNAs that mediate intercellular contact between macrophages (U937 cells) and human umbilical vein endothelial cells (HUVECs), and further explore the detailed molecular mechanisms involved, which may contribute to the pathological processes leading to atherosclerosis.

## 2 MATERIALS AND METHODS

### 2.1 Cell culture

HUVECs and U937 cells were both purchased from Procell Life Science & Technology (Procell), and maintained separately in Endothelial Cell Medium (Sciencell) and RPMI‐1640 supplemented with 10% foetal bovine serum, respectively. HEK293T cells were also purchased from Procell Life Science & Technology and were maintained in high glucose DMEM supplemented with 10% fetal bovine serum. All cells were cultured in a cell incubator with 5% CO_2_ at 37°C.

### 2.2 Preparation of foam cell models

Macrophages differentiation from U937 cells in vitro were induced by treatment with 100 ng/ml PMA (Selleck, S7791) for 24 h, and macrophage‐derived foam cell model was generated by incubation with 100 μg/mL ox‐LDL for 24 h.

### 2.3 Co‐culture of U937 macrophages with HUVECs

5 × 10^5^ cells of U937 macrophages and HUVECs were seeded separately into the upper and lower chambers of six‐well Transwell plates (Corning, 3412) and GW4869 was used to block macrophage exosome secretion. After co‐culture for 24 h, levels of miR‐4532 expression were measured in U937 cells, HUVECs and co‐culture supernatants, as well as ICAM and VCAM in HUVECs was detected by qRT‐PCR and Western blot analyses.

### 2.4 Oil red O staining

After treatment of U937 cells with ox‐LDL or HUVECs with exosomes, cells were stained with oil red O (Solarbio, G1262), which appeared as orange‐red or pink oil droplets in the cytoplasm. Images were captured using an Olympus microscope (Olympus‐BX530).

### 2.5 Collection and identification of exosomes

Five millilitres of whole blood were collected from each patient in EDTA tubes (BD biosciences). Exosomes were isolated and purified from plasma by ultracentrifugation. After mixed plasma with PBS, mixture was centrifuged at 2000 × *g* for 10 min, then the supernatant was collected and centrifuged at 12,000 × g for 45 min. The supernatants were then passed through a 0.22 μm filter (Millipore, GVWP02500) and ultracentrifuged at 110,000 × *g* for 90 min. The pellets were then washed with phosphate‐buffered saline (PBS) followed by the second ultracentrifugation at 110,000 × *g* for 90 min. After ultracentrifugation, we used 100 μl of PBS to resuspend the pellet. All above procedures were performed at 4°C. Following ultracentrifugation, supernatants were used for exosomes extraction. Transmission electron microscopy (Hitachi HT7700) and Nanoparticle Tracking Analysis (ZetaView) were used to determine exosome purity and concentration. Further, the exosome protein markers, Hsp70 (CST, #4876, 1:1000), TSG101 (Santa Cruz Biotechnology, sc‐7964, 1:500), and CD9 (CST, #13174, 1:1000), and the negative marker, calnexin (Santa Cruz Biotechnology, sc‐23,954, 1:500), were used to identify exosomes by Western blotting.

### 2.6 Exosome uptake assay

After treatment of U937 cells with human ox‐LDL (Yiyuan Biotechnologies Guangzhou) for 24 h, cell supernatants were collected and the exosomes was extracted similar to the extraction of plasma exosomes. 100 μl exosomes were incubated with 100 μl of PKH‐67 dye (BestBio) for 30 min at 37°C in the dark, then added into HUVECs cell medium for 3 h. Subsequently, HUVECs were fixed with 4% paraformaldehyde and stained with DAPI. Images were acquired by confocal microscopy (Leica Confocal Laser Scanning Microscope SP8).

### 2.7 Patient recruitment

Patients were recruited from Central Hospital Affiliated with Shandong First Medical University between June 2019 and July 2021. There were no significant differences in age or sex between the two study groups. Participants included three diagnosed with acute myocardial infarction (AMI) and three healthy controls. Subsequently, plasma exosomes from 42 patients with AMI, 14 with unstable angina (UA) and 22 healthy controls were subjected to RT‐qPCR for secondary validation. This study was approved by the ethics committee of Jinan Central Hospital (No. 2018–039‐01). The clinical characteristics and summary statistics of subjects are described in Tables 1 and 2.

**TABLE 1:** Characteristics of patients

Variable	Control (*n* = 22)	UA (*n* = 14)	AMI (*n* = 42)	*p* value
Age (y)	57.09 ± 8.09	63.71 ± 10.42	61.24 ± 9.92	0.105
LVEF (%)	NA	61.9231 ± 5.18751	54.0952 ± 6.59567	0.0003
E Peak	NA	65 ± 17.05872	52.25 ± 9.84463	1.264
A peak	NA	87.3333 ± 22.03028	83.25 ± 24.00521	0.2301
HR (beats/min)	77.32 ± 12.32	80.79 ± 11.71	76.86 ± 10.16	0.511
TG (mmol/L)	1.43 ± 0.64	1.42 ± 0.7	1.43 ± 0.75	0.998
TC (mmol/L)	4.22 ± 0.91	4.23 ± 1.15	4.55 ± 0.99	0.377
LDL (mmol/L)	2.52 ± 0.79	2.52 ± 0.96	2.79 ± 0.71	0.32
HDL (mmol/L)	1.15 ± 0.22	1.12 ± 0.2	1.2 ± 0.51	0.802

*Note*: Data are expressed as the means ± SD. Statistical analysis was carried out using one‐way anova.

Abbreviations: AMI, acute myocardial infarction; UA, unstable angina pectoris;LVEF, left ventricular ejection fraction; E Peak, Maximum early left ventricular diastolic flow; A Peak, maximum mitral atrial systolic flow.

**TABLE 2:** Summary of medication use

Drugs/Groups	UA (*n* = 14) (Preoperative)	UA (*n* = 14) (Postoperative)	AMI (*n* = 42) (Preoperative)	AMI (*n* = 42) (Postoperative)
Aspirin	2	12	4	42
Tegretol	NA	6	NA	33
Statins	2	11	NA	38
Beta blockers	4	8	1	26
Ezetimibe	NA	2	NA	5
Proton pump inhibitors	NA	7	NA	28
Isosorbide mononitrate	3	2	NA	3
ACEI/ARB	3	7	3	24
Trimetazidine	1	1	1	NA
CCB	4	3	3	1
Clopidogrel	NA	4	NA	12
Diuretics	NA	2	NA	5
Ivabradine	2	12	NA	15

Abbreviations: ACEI/ARB, Angiotensin‐converting enzyme inhibitors/angiotensin receptor antagonists; CCB, Calcium channel blockers.

The inclusion criteria for patients with AMI were as follows:

- Age 35–75 years.
- No smoking history or quit smoking for more than 3 months.
- Chest pain lasting more than 30 min with no relief; time window was within 12 h from the start of chest pain, including both acute ST elevation and non‐ST elevation myocardial infarction.
- At least one time‐point with troponin T or troponin I above the upper normal limit (99th percentile of the upper reference value).
- Patients with ≥70% stenosis of the affected vessel identified by coronary angiography, which is required for percutaneous coronary intervention (PCI).
- No anticoagulants were used.
- Systolic blood pressure level 90–140 mmHg, and diastolic blood pressure <90 mmHg (patients with a history of well‐controlled hypertension were also enrolled).

The inclusion criteria for patients with UA patients were that they should both meet the diagnostic criteria for unstable angina pectoris and a, b, e, f and g above.

### 2.8 Exosomal miRNA sequencing analysis

To further explore the mechanism mediated by macrophage‐derived exosomes, we firstly extracted exosomes from plasma. Then, total RNA was extracted from exosomes using the miRNAs serum/plasma kit (Qiagen, 217,184). The process of sequencing was fully controlled by the data collection software provided by Illumina Xten (Illumina), and the sequence results were collected and analysed by data collection software. Target genes of differentially expressed miRNAs were predicted by the TargetScan 8.0 software. Gene ontology (GO), Kyoto Encyclopedia of genes and genomes (KEGG) enrichment analyses were performed on DAVID 6.8 online databases (https://david.ncifcrf.gov). And the ggplot2 package (version 3.3.5) in R language (version 4.0.5) was adopted to draw bar and bubble plots for visualization, respectively.

### 2.9 Cell transfection

Lipofectamine 2000 (Thermo Fisher Scientific) was used to transfect U937 cells or HUVECs with 10 nmol/L of the miR‐4532 inhibitor or mimic (RiboBio), according to the manufacturer's instructions. To identify the role of the target protein, SP1, siRNAs (RiboBio) and pcDNA3.1‐SP1 (Tsingke) were used to transfect HUVECs, following detection of p‐eNOS, ET‐1, VCAM‐1 and ICAM‐1 levels in HUVECs.

### 2.10 RNA isolation, reverse transcription and real‐time PCR

Trizol was used to isolate total RNA and an Evo M‐MLV RT Mix Kit to generate cDNA. Cellular miRNAs were extracted using AG RNAex Pro Reagent, all reagent in this procedure were purchased from Accurate Biology (Changsha). Exosomal miRNAs were extracted using miRNAs Serum/Plasma kit, and cDNA was synthesized according to the instructions (Accurate Biology). RT‐qPCR was performed on a Real‐Time PCR System using the SYBR Green Premix Pro Tag HS qPCR kit (Accurate Biology). Relative levels of each mRNA were normalized to those of GAPDH, and miRNA levels were normalized to those of U6. All primers sequences are listed in Table 3.

**TABLE 3:** Primer sequences

MicroRNAs or gene name	Primer sequence (5′ to 3′)
hsa‐miR‐4532‐forward	CCCCGGGGAGCCCGGCG
hsa‐miR‐1‐3p‐forward	UGGAAUGUAAAGAAGUAUGUAU
hsa‐miR‐30b‐5p‐forward	UGUAAACAUCCUACACUCAGCU
hsa‐miR‐144‐3p‐forward	UACAGUAUAGAUGAUGUACU
hsa‐miR‐203a‐3p‐forward	GUGAAAUGUUUAGGACCACUAG
hsa‐U6‐forward	CTCGCTTCGGCAGCACA
hsa‐U6‐reverse	AACGCTTCACGAATTTGCGT
hsa‐ICAM‐1‐forward	AGCTTCGTGTCCTGTATGGC
hsa‐ICAM‐1‐reverse	TTTTCTGGCCACGTCCAGTT
hsa‐VCAM‐1‐forward	AATTCCACGCTGACCCTGAG
hsa‐VCAM‐1‐reverse	GGCCACCACTCATCTCGATT
hsa‐SP1‐forward	CAGGACCCCCTTGAGCTTGTC
hsa‐SP1‐reverse	CCTGTTCCCCCTGACTGACT
hsa‐GAPDH‐forward	CCGTTGAATTTGCCGTGA
hsa‐GAPDH‐reverse	TGATGACCCTTTTGGCTCCC

### 2.11 Western blot

Briefly, total intracellular proteins were extracted using protein lysis buffer (1% SDS, 25 mM Tris–HCl [pH 7.5], 4 mM EDTA, 100 mM NaCl, 1 mM PMSF, 10 mg/ml leupeptin, and 10 mg/ml soybean trypsin inhibitor). Then, protein samples were separated by 10% SDS‐PAGE and transferred onto PVDF membranes (Millipore). Membranes were incubated with primary antibodies specific for rabbit p‐eNOS (S1177) (Abcam, ab215717, 1:1000), eNOS (Proteintech, 27,120‐1‐AP, 1:1000), ET‐1 (Proteintech, 12,191‐1‐AP, 1:1000), ICAM‐1 (Proteintech, 10,831‐1‐AP, 1:1000), mouse VCAM‐1 (Proteintech, 66,294‐1‐lg, 1:1000), rabbit p‐P65 (CST, #3033, 1:1000), mouse P65 (Santa Cruz Biotechnology, sc‐8008, 1:1000), and rabbit‐SP1(Proteintech, 21,962‐1‐AP, 1:1000) These proteins were then incubated with a horseradish peroxidase‐conjugated IgG. Band intensities were quantified using ImageJ software (National Institutes of Health) and normalized to β‐actin levels.

### 2.12 Actinomycin D assay

HUVECs cells were divided into negative control, mimic and inhibitor groups. After 24 h of transfection, actinomycin D (5 μg/ml; Millipore) was added to the culture medium and RNA was collected at 0, 2, 4 and 6 h and used for detection of SP1 mRNA levels by RT‐qPCR.

### 2.13 Dual‐luciferase reporter gene assay

Wild‐type and mutated miR‐4532 binding sites in the 3′UTR of SP1 were cloned into the pmir‐GLO luciferase vector to generate the plasmids, pmirGLO‐hSP1‐WT and pmirGLO‐hSP1‐MUT, respectively, were by BioSune. HEK293T cells were transfected with miR‐4532 mimic and indicated plasmids using Lipofectamine 2000. After 48 h incubation, luciferase activity was quantified using a dual‐luciferase reporter assay kit (Promega) on a Centro LB 963 luminometer (Berthold Technologies).

### 2.14 RNA immunoprecipitation assay

RNA immunoprecipitation (RIP) assays were performed using the RIP Kit (Genesee Biotech, Cat. No. P0101), following the manufacturer's instructions. In brief, 1 × 10^7^ cells were cleaved in RIP lysis buffer and then incubated with magnetic beads containing anti‐argonaute‐2 (AGO2) antibody (Millipore, #03–110) or normal mouse IgG (Millipore, #03–110) overnight at 4°C. SP1 and miR‐4532 levels of were analysed by RT‐qPCR.

### 2.15 Statistical analysis

All data are expressed as mean ± standard error. SPSS 22.0 statistical software and GraphPad Prism 8.0 were used for statistical analyses, including Student's two‐tailed t‐test and chi‐square test. Multiple comparisons between groups were performed using anova. *p* < 0.05 was considered statistically significant.

## 3 RESULTS

### 3.1 Secretion of exosomes by macrophages affects HUVEC function

**FIGURE 1:**
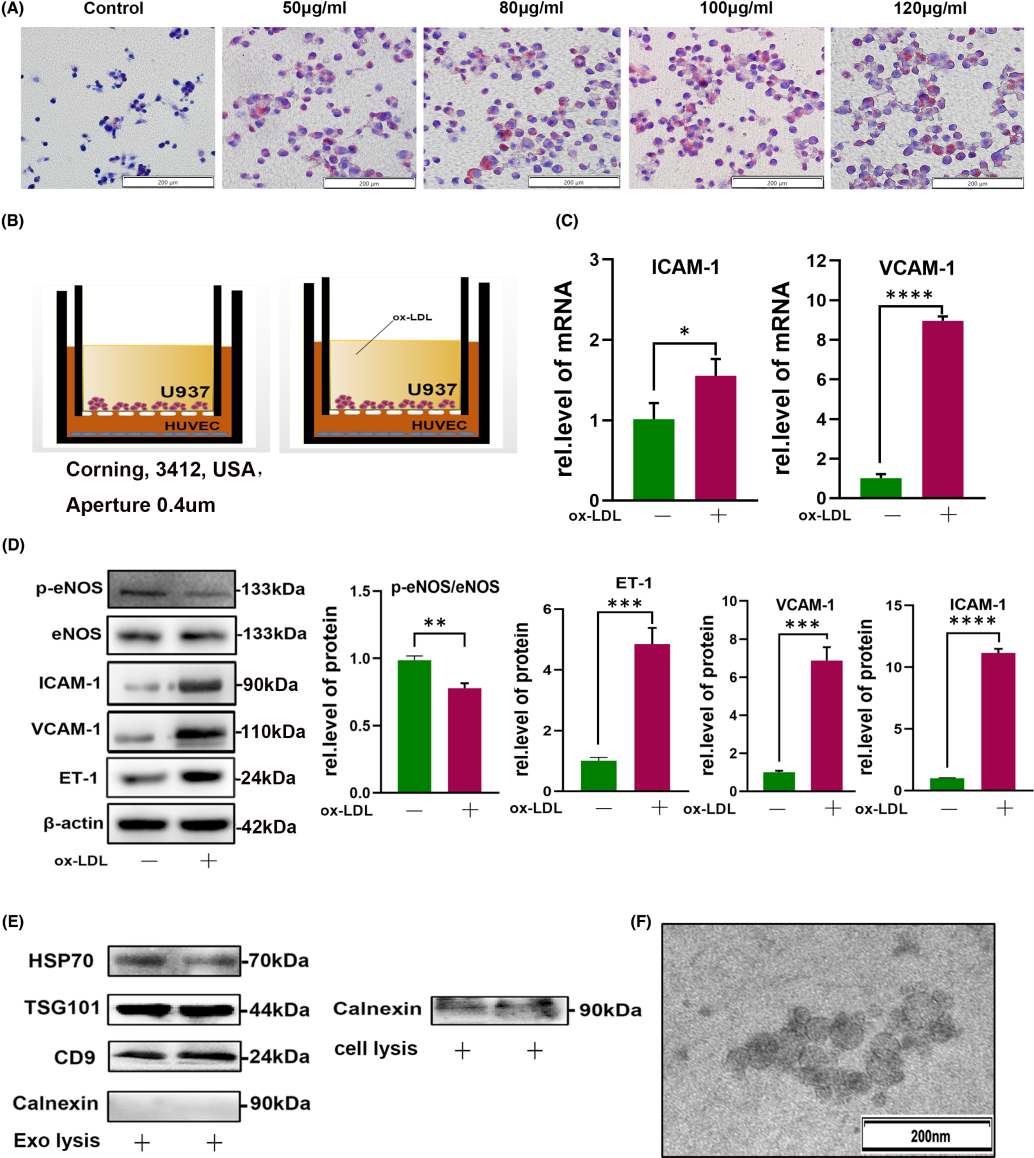
U937 macrophages stimulated by ox‐LDL affect HUVEC function. (A) U937 macrophages stimulated with different concentrations of ox‐LDL and red lipid droplets in the cytoplasm stained with oil red O. Scale bars, 200 μm. (B) Diagram illustrating the system for transwell co‐culture of U937 and HUVECs. (C, D) After ox‐LDL stimulation, the relative expression levels of p‐eNOS, ET‐1, ICAM‐1 and VCAM‐1 in HUVECs were analysed by ImageJ software. (E, F) Protein markers and transmission electron microscopy of exosomes in cell supernatants. *p < 0.05, **p < 0.01, ***p < 0.001, and ****p < 0.0001

After induction of macrophages with 100 nM PMA, ox‐LDL (50, 80, 100 or 120 μg/ml) was added into U937 cell culture medium. Following oil red O staining analysis, 100 μg/ml ox‐LDL was selected to generate foam cells (Figure 1A). Next, we further study the function of exosomes on endothelial. After co‐culture of macrophages and HUVECs (Figure 1B), functional molecules, including ET‐1, ICAM‐1 and VCAM‐1 levels increased and p‐eNOS decreased significantly after ox‐LDL treatment, relative to untreated controls (Figure 1C,D).

### 3.2 Exosomes mediate intercellular communication between macrophages and HUVECs

**FIGURE 2:**
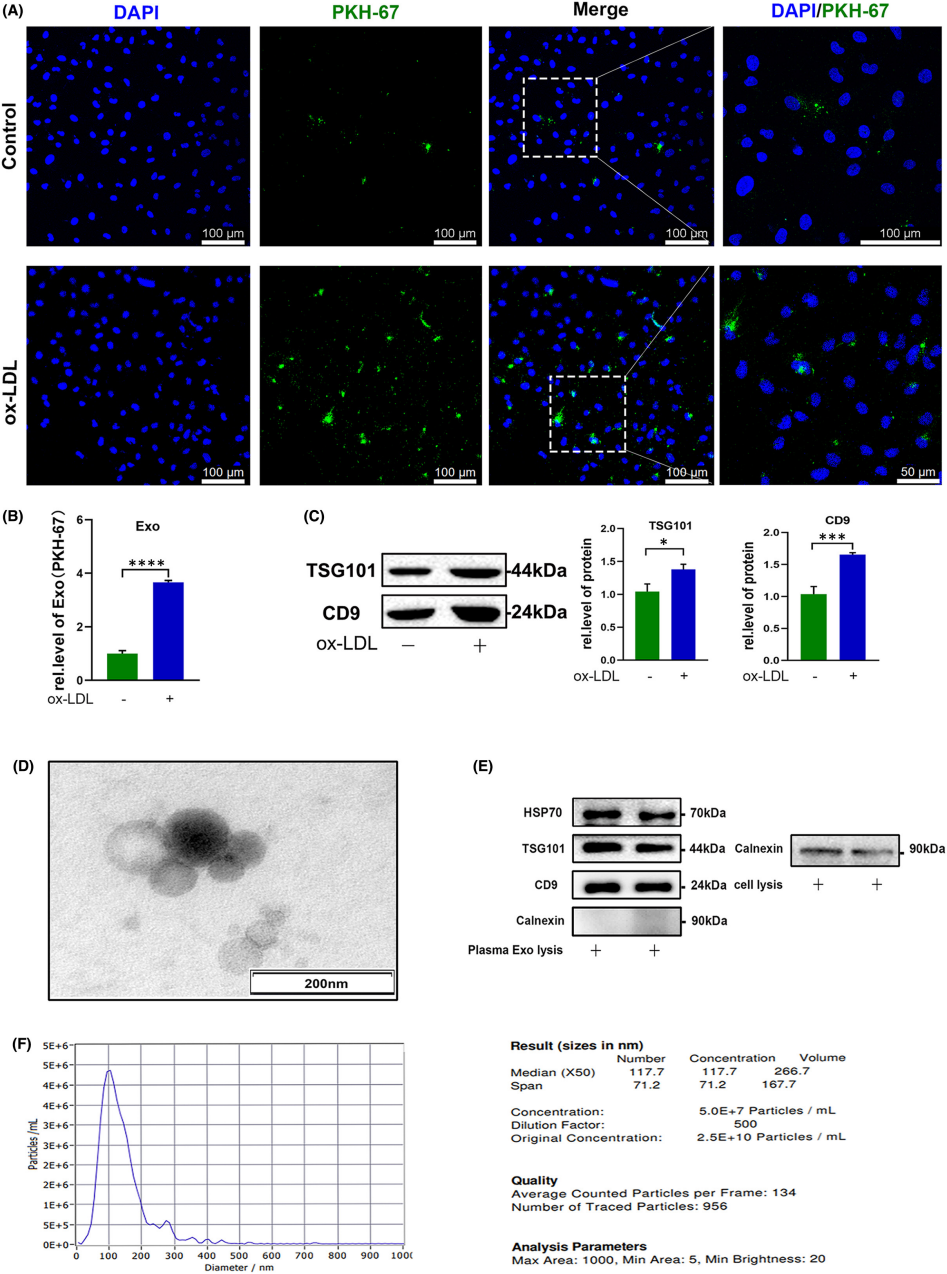
Exosome uptake assay and identification of exosomes in plasma. (A, B) HUVECs take up exosomes from U937 macrophage culture supernatant; exosomes were labelled with PKH‐67 and analysed by ImageJ software. Scale bars, 100 μm. (C) Exsome marker, TSG101 and CD9 were measured by Western blot to partly quantify the levels of exosome internalization in both conditions. (D–F) Exosomes were extracted from human plasma and identified by transmission electron microscopy, Western blot, and nanoparticle tracking analysis

Exosomes are small membrane vesicles (30–150 nm), which play vital roles in intercellular communication. We hypothesized that macrophage‐derived exosomes may mediate macrophage influence on HUVECs. Exosomes were successfully extracted from medium, as confirmed by Western blot (shown as two repeat lanes) and TEM analyses (Figure 1E,F). Ox‐LDL treatment significantly increased the density of green dots in HUVECs, indicating that it induced exosome uptake by these cells (Figure 2A–C), providing support for our hypothesis.

### 3.3 Exosomal miRNA sequencing analysis

**FIGURE 3:**
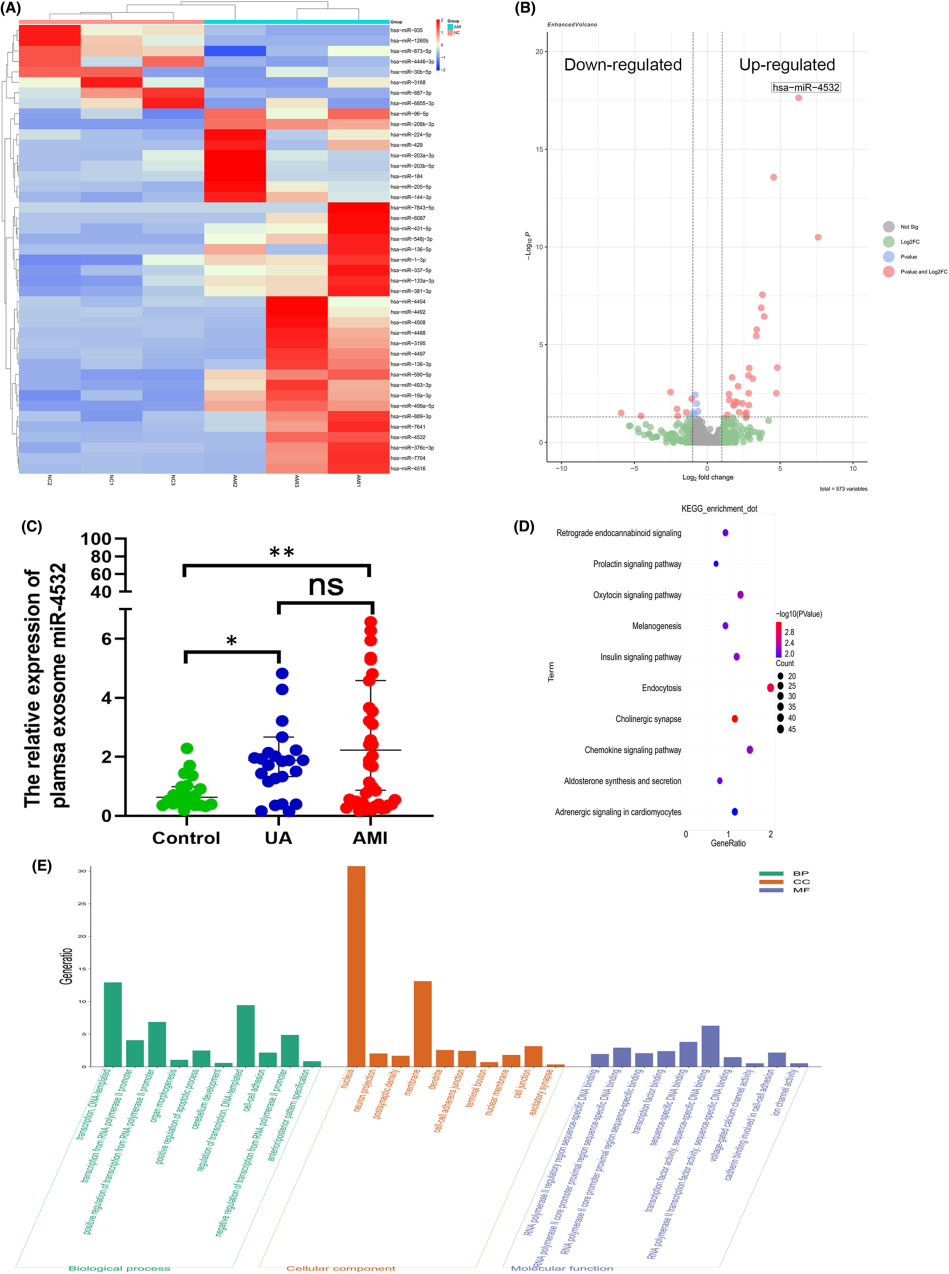
RNA‐sequence analysis of miRNAs from plasma exosomes. (A) Heatmap of 43 differentially expressed miRNAs. (B) Volcano map illustrating differentially expressed miRNAs; bubbles in upper left quadrant and upper right quadrant represent down‐regulated and up‐regulated miRNAs, respectively. (C) Clinical plasma samples were collected, and the expression level of miR‐4532 in different groups determined by qRT‐PCR. (D, E) KEGG pathway and GO analysis of mi‐4532 predicted target genes. *p < 0.05 and **p < 0.01

Atherosclerosis is a common cause of AMI. To further clarify the mechanism underlying this process, we extracted plasma exosomes from 3 patients with AMI and 3 healthy controls (Figure 2D–F) and performed miRNA sequencing. Plotting a heatmap of levels of all detectable miRNAs in both the AMI and control groups indicated that 30 miRNAs were up‐regulated and 7 were down‐regulated in AMI relative to healthy controls(∣log2fold Change∣ ≥ 1 and *p* < 0.05; Figure 3A,B).

### 3.4 Plasma exosomal miR‐4532 is increased in patients with UA and AMI

To further confirm our sequencing results, we extracted plasma exosomes from 42 patients with AMI, 14 with UA, and 22 healthy controls, to verify the differential expression of miR‐1, miR‐30, miR‐144, miR‐208 and miR‐4532 levels in both the AMI and control groups were tested to verify the sequencing result for their more obvious up‐regulation and abundance by qRT‐PCR. Interestingly, we found that miR‐4532 levels were significantly higher in both AMI and UA plasma exosomes than those in the healthy control group (Figure 3C) versus other miRNA candidates (Figure S1A). Further, KEGG analysis of miR‐4532 target genes indicated that endocytosis was the most relevant enriched pathway (Figure 3D). Further, GO enrichment analysis suggested that miR‐4532 is closely related to cell junction and transcription factor binding (Figure 3E).

### 3.5 Exosomes mediate miR‐4532 transfer from macrophages to HUVECs

**FIGURE 4:**
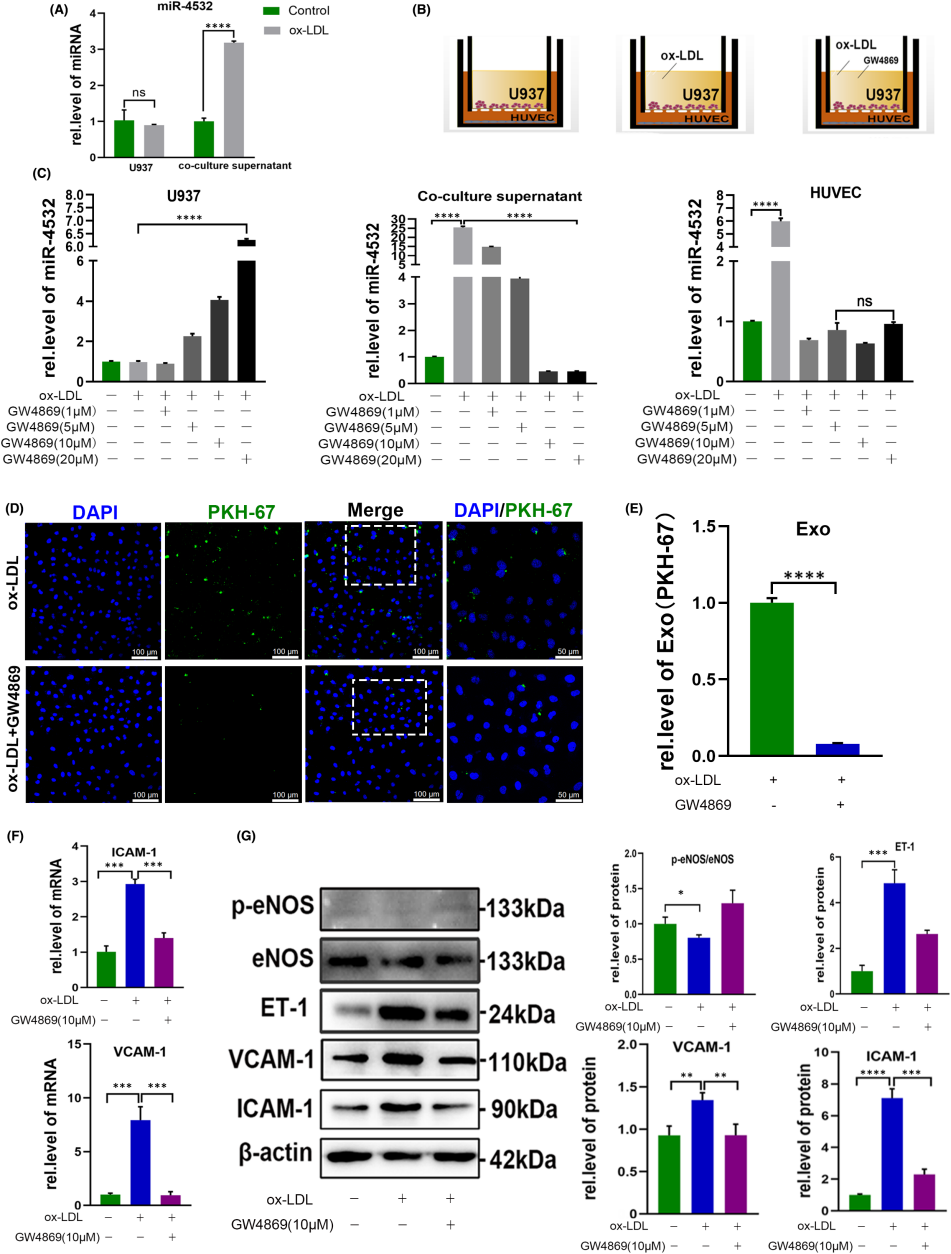
GW4869 inhibited exosome secretion and ameliorated HUVEC dysfunction. (A) Relative expression level of miR‐4532 in U937 macrophages and supernatants from the transwell co‐culture system. (B) Diagram illustrating the transwell co‐culture system under different treatments, exosomes from U937 macrophages, supernatants and HUVECs were collected. (C) U937 macrophages in the upper chamber were treated with different concentrations of GW4869 (1, 5, 10, and 20 μM), with or without ox‐LDL, and the relative expression levels of miR‐4532 in U937 macrophages, co‐culture supernatants, and HUVECs tested by qRT‐PCR and Western blot analyses. (D, E) Exosomes uptake assay following treatment with ox‐LDL in the presence or absence of GW4869. (F, G) Relative expression levels of p‐eNOS, ET‐1, ICAM‐1 and VCAM‐1 in HUVECs after treatment of U937 cells with GW4869. *p < 0.05, **p < 0.01, ***p < 0.001, and ****p < 0.0001

First, we detected exosomal miR‐4532 levels in both U937 cells and culture supernatants. Ox‐LDL treatment resulted in miR‐4532 accumulation in the culture medium, rather than in the cell cytoplasm (Figure 4A, Figure S1B). Next, we treated cells with the exosome inhibitor, GW4869 in different concentration, to validate the role of exosomes in intercellular communication (Figure 4B). Our results showed that GW4869 could inhibit exosome secretion from U937 cells to the culture medium and take up by HUVECs in a dose‐dependent manner (Figure 4C). Further, an exosome uptake assay also demonstrated that GW4869 inhibited exosome uptake by HUVECs (Figure 4D,E), resulting in decreased ET‐1, ICAM‐1, VCAM‐1 and increased p‐eNOS levels (Figure 4F,G).

### 3.6 Overexpression or knockdown of miR‐4532 in U937 cells influences the exosome‐mediated functions of HUVECs

**FIGURE 5:**
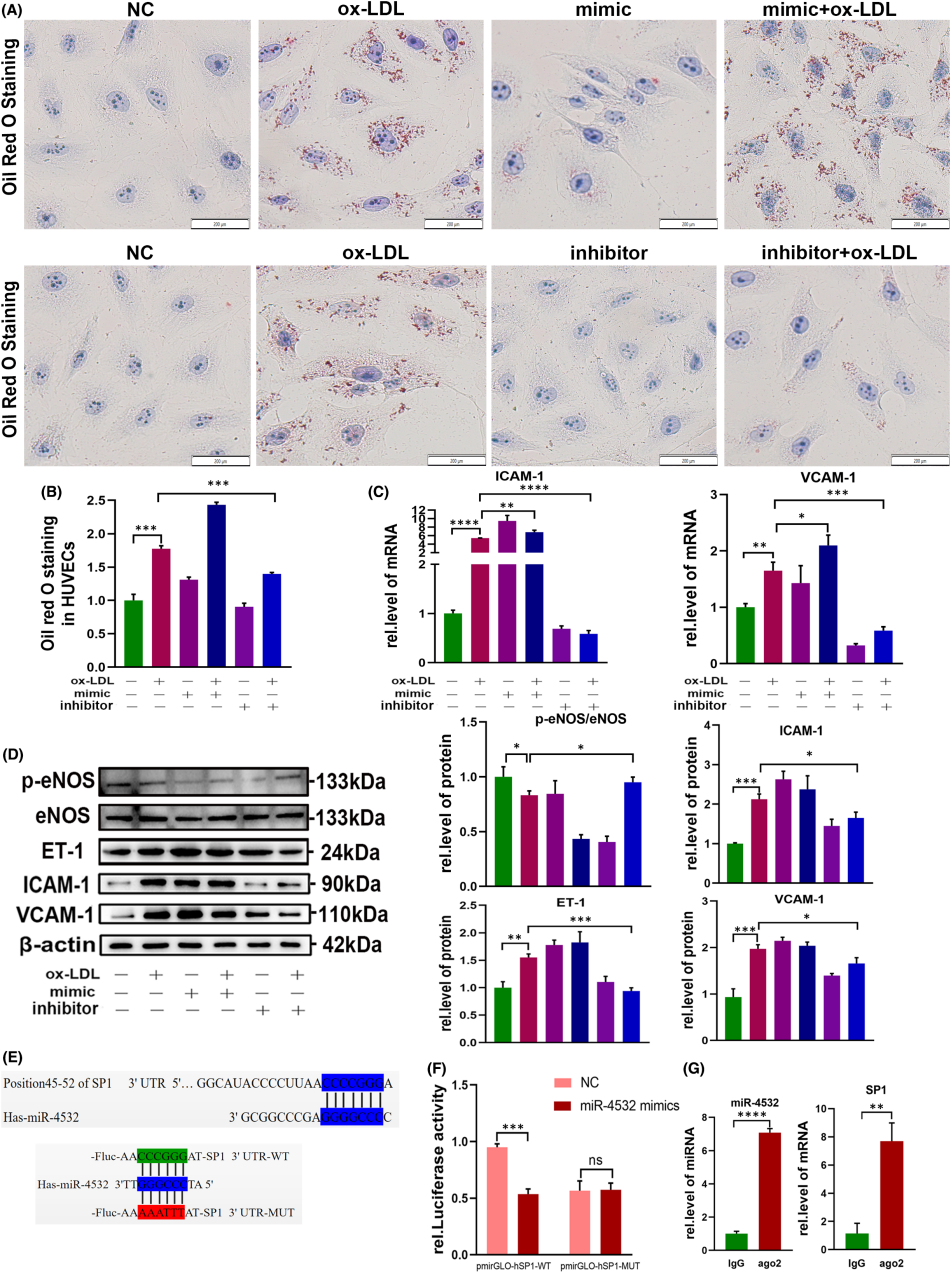
Exosomal miR‐4532 derived from U937 macrophages promotes HUVEC disruption, possibly through targeting SP1. (A, B) After transfection of miR‐4532 mimic/inhibitor into U937 macrophages, oil red O staining was used to visualize lipid droplets within HUVECs and analysed by ImageJ. Scale bars, 200 μm. (C, D) Relative expression levels of p‐eNOS, ET‐1, ICAM‐1 and VCAM‐1 in HUVECs following transfection of U937 macrophages with miR‐4532 mimic/inhibitor. (E) Base pairing sites between miR‐4532 and the 3′‐UTR of SP1 predicted using TargetScan. (E) miR‐4532 binding sites in the 3′UTR of SP1 were cloned into the pmir‐GLO luciferase vector. (F) Effect of transfection of miR‐4532 mimic on activity of luciferase reporter constructs containing wild‐type (WT) or mutant (MUT) SP1 3′‐UTR. (G) RIP performed in HUVECs transfected with miR‐4532 mimic. SP1 and miR‐4532 were detected by qRT‐PCR. IgG served as a negative control for RIP. *p < 0.05, **p < 0.01, ***p < 0.001, and ****p < 0.0001

We transfected U937 cells with miR‐4532 mimic or inhibitor to further confirm our hypothesis. Transfection efficiency of miR‐4532 mimic or inhibitor in U937 cells were tested by qRT‐PCR (Figure S1C). The result showed that treatment with miR‐4532 mimic increased the numbers of oil red O droplets in HUVECs compared with treatment with ox‐LDL alone, while miR‐4532 inhibitor antagonized the effect of ox‐LDL (Figure 5A,B). Consistent with these findings, p‐eNOS, ET‐1, ICAM‐1 and VCAM‐1 expression levels in HUVECs from the mimic + ox‐LDL group were significantly altered compared with those in the ox‐LDL group, while miR‐4532 inhibitor reversed the effects of ox‐LDL (Figure 5C,D). Overall, these data demonstrate that miR‐4532 has a vital role in macrophage‐derived exosome transfer to, and disruption of, HUVECs.

### 3.7 MiR‐4532 promotes atherosclerosis by targeting SP1

**FIGURE 6:**
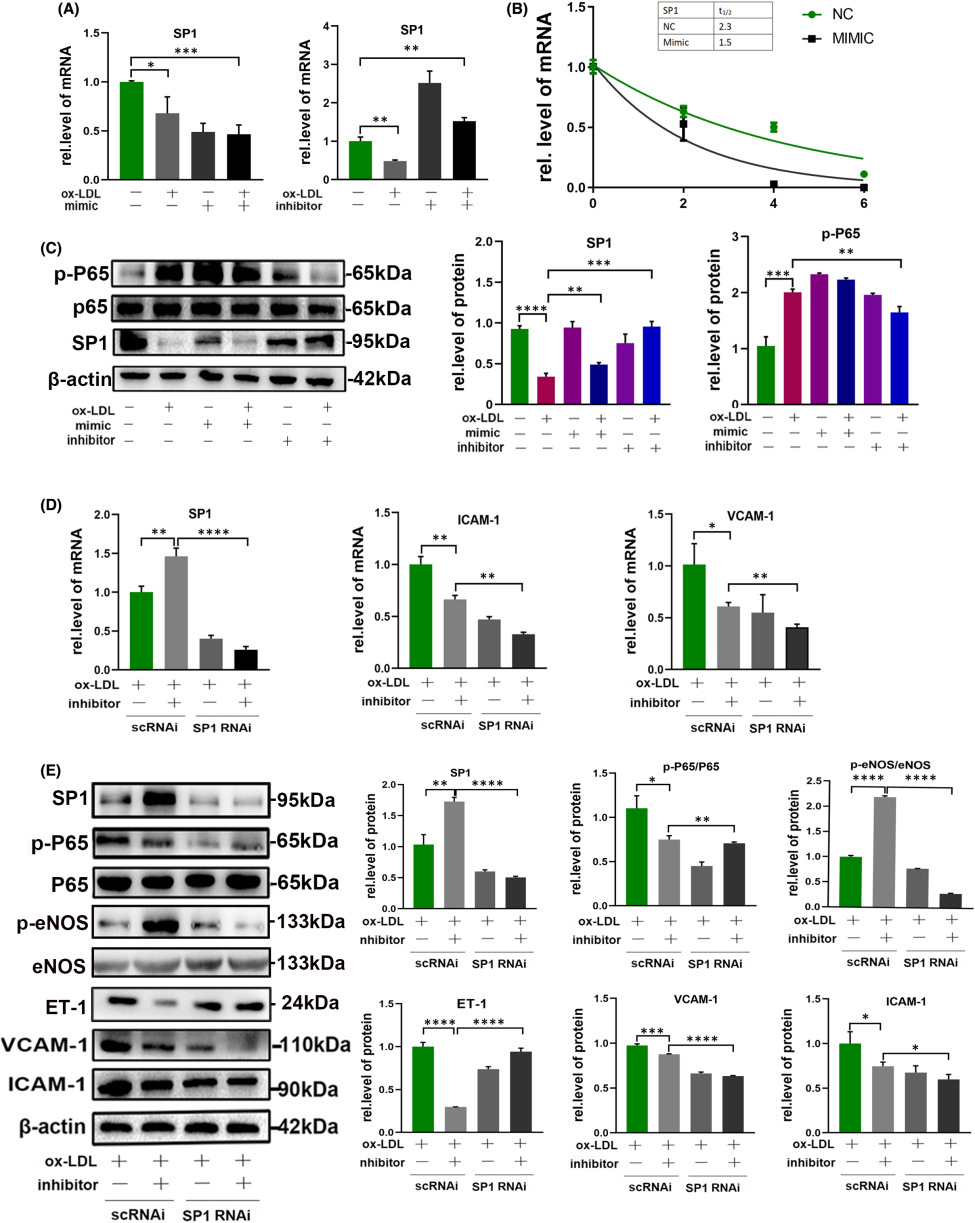
Transfection of U937 cells with miR‐4532 affected HUVEC function through targeting SP1. U937 macrophages were transfected with miR‐4532 mimic/inhibitor. (A) qRT‐PCR was used to evaluate SP1 mRNA expression levels. (B) SP1 transcription was inhibited by actinomycin D, and SP1 mRNA levels in HUVECs detected by qRT‐PCR at specific time points. (C) Relative SP1 and p‐P65 protein expression levels were visualized by Western blot and analysed using ImageJ. HUVECs were transfected with SP1‐specific siRNA. (D, E) Relative expression levels of ICAM‐1, VCAM‐1, SP1, and p‐P65 mRNA and protein was measured by qRT‐PCR and Western blot. *p < 0.05, **p < 0.01, ***p < 0.001, and ****p < 0.0001

To determine the molecular mechanism underlying the effects of miR‐4532 in macrophage‐HUVEC communication, we chose SP1 as a potential target of miR‐4532, based on TargetScan analysis and previous literature reports (Figure 5E).^29^, ^30^, ^31^ Luciferase reporter and RIP assays were then used to confirm the targeting relationship between miR‐4532 and SP1. Co‐transfection of miR‐4532 mimic and pmirGLO‐hSP1‐WT led to significantly decreased luciferase activity relative to the control group, while there was no obvious change in the luciferase activity of cells co‐transfected with miR‐4532 mimic and pmirGLO‐hSP1‐MUT (Figure 5F). Next, we performed an RIP assay, to confirm the interaction between miR‐4532 and SP1. Ago2 antibody was used to pulldown its binding RNA and protein, and SP1 and miR‐4532 were significantly enriched in the ago2‐pulldown group (Figure 5G). Further, mimic miR‐4532 treatment decreased SP1 mRNA levels, while miR‐4532 inhibitor reversed this effect (Figure 6A). Next, actinomycin D was used to inhibit SP1 mRNA synthesis, and miR‐4532 mimic treatment increased the half‐life of SP1 (Figure 6B). These results indicate that an RNA‐induced silencing complex loaded with miR‐4532 binds directly to the 3’UTR of SP1 and induces its degradation.

### 3.8 Degradation of SP1 disrupts HUVECs by activating NF‐κB P65

**FIGURE 7:**
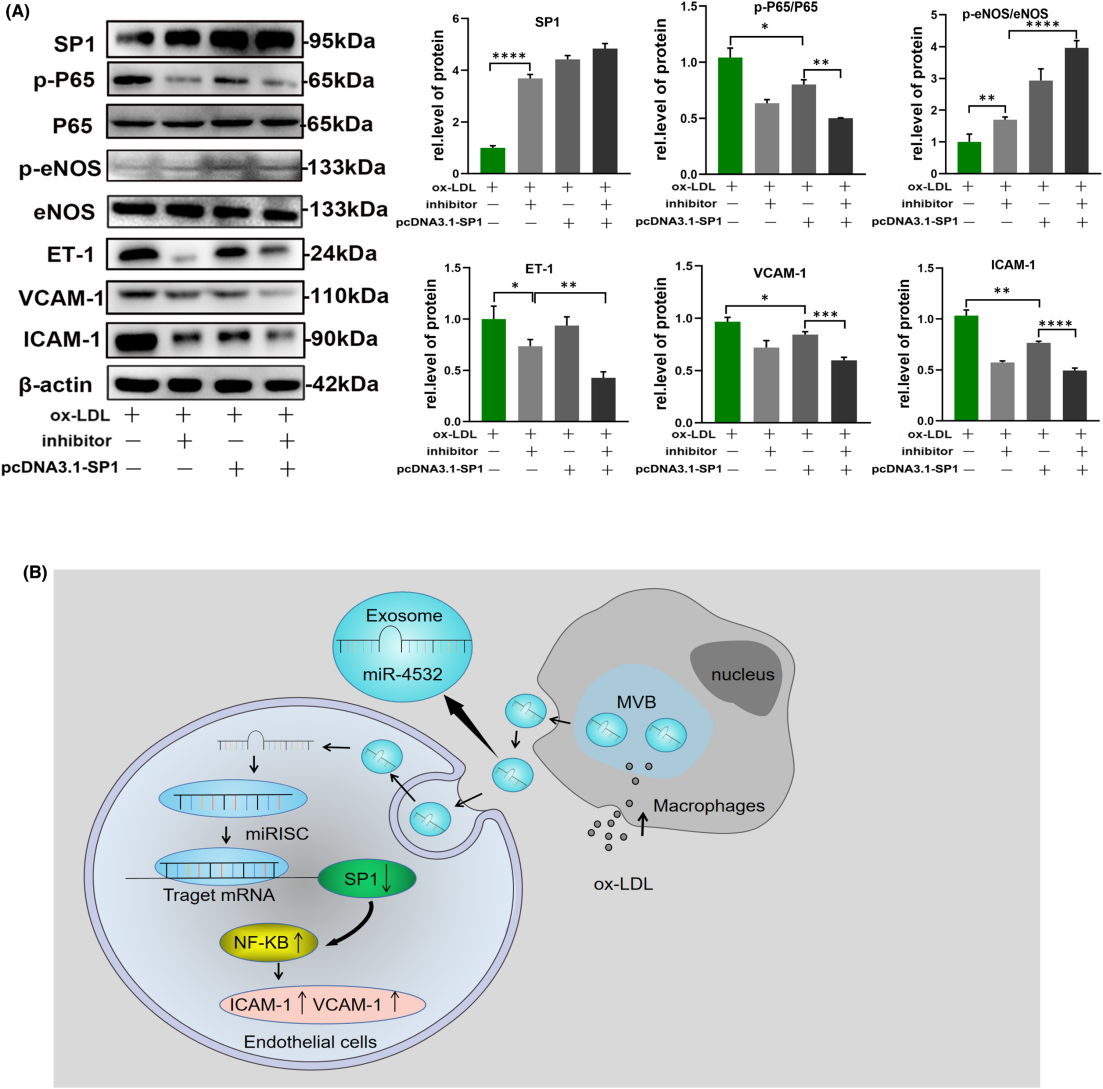
Over expression of SP1 alleviated the role of macrophages‐derived exosomal miR‐4532 in HUVECs and the schematic diagram of exosomal miR‐4532 disruption of HUVECs through targeting SP1. (A) After transfection of HUVECs with pcDNA3.1‐SP1 plasmid, NF‐κB P65, ET‐1, ICAM‐1 and VCAM‐1 of it obviously decreased, while p‐eNOS level was increased compared to negative control groups. (B) U937 macrophages transform into foam cells under stimulation with ox‐LDL, which promotes exosomes secretion and their uptake by HUVECs. Exosomal miR‐4532 targets and destabilizes SP1 mRNA, which results in SP1 reduction, resulting in NF‐κB P65 activation and HUVECs disruption

NF‐κB is the key translation factor involved in VCAM‐1 and ICAM‐1 regulation. Searches of relevant articles indicated that SP1 can regulate NF‐κB activation in cancer.^32^ Relative SP1 mRNA and protein expression levels were lower in the ox‐LDL + mimic and mimic groups than those in the control group, and treatment with inhibitor reversed the effect of ox‐LDL. Interestingly, p‐P65 presented the opposite trend to SP1, partly supporting our hypothesis (Figure 6C). To further explore these findings, we knocked down SP1 in HUVECs. The result showed that ET‐1, ICAM‐1 and VCAM‐1 expression levels were relatively decreased and following the increased p‐eNOS level in the inhibitor + ox‐LDL group compared with the ox‐LDL group, while SP1 knockdown abolished the effect of ox‐LDL in activating NF‐κB P65 and disrupting HUVECs function (Figure 6D,E, Figure S1D,E). In other words, miR‐4532 lost the effect on HUVECs in absence of SP1. Correspondingly, overexpression of SP1 by transfecting HUVECs with pcDNA3.1‐SP1 plasmid obviously alleviated the effect of ox‐LDL in activating NF‐κB P65, following the alteration of functional markers of HUVECs (Figure 7A, Figure S1F–H). All these data demonstrated that SP1 was the indispensibe mediator in miR‐4532 effects on HUVECs.

## 4 DISCUSSION

Atherosclerosis is a complex process, involving interactions among endothelial cells, immune cells and vascular smooth muscle cells.^33^ At present, treatments for atherosclerosis mainly focus on early intervention to address various risk factors, to stabilize atherosclerotic plaques and prevent target organ infarction.^34^, ^35^ Ox‐LDL stimulates endothelial cells to release proinflammatory cytokines and chemokines, thereby promoting the aggregation of white blood cells, such as monocytes and T cells, on the surface of endothelial injury, resulting in endothelial cell apoptosis and endothelial dysfunction.^36^, ^37^ The term ‘endothelial barrier’ refers to the selective permeability barrier between blood vessel walls and blood comprising endothelial cells. Endothelial dysfunction is generally considered the initial step in vascular endothelial cell barrier dysfunction, and disturbance of endothelial barrier function promotes inflammation and accelerates atherosclerotic plaque formation.^38^, ^39^, ^40^, ^41^ Recently findings suggest that different cell types can secrete or receive exosomes containing miRNAs and that these play important roles in regulating cellular functions.^42^ Our data indicate that macrophage‐derived exosomes can alter endothelial cell function.

In the present study, by sequencing miRNAs in plasma exosomes from patients with AMI, we found differential expression of multiple miRNAs. Based on the combined analysis of fold change and *p*‐value, the most prominent of which was miR‐4532. GO and KEGG analyses indicated that miR‐4532 is involved in multiple biological functions and signalling pathways, including cell–cell adhesion, transcription, endocytosis, insulin secretion signalling and chemokine signalling, among others. In subsequent experiments, we discovered a novel mechanism, where ox‐LDL induced U937 macrophages to become foam cells, up‐regulated macrophage miR‐4532 expression, and then macrophage‐derived miR‐4532 was transferred to endothelial cells via exosomes, resulting in endothelial cell dysfunction.

According to TargetScan software analysis, SP1was predicted to be the target protein of miR‐4532; therefore, we conducted dual‐luciferase and RIP experiments to determine whether miR‐4532 could inhibit expression of the SP1 protein and found that transduction with macrophage‐derived miR‐4532 exosomes effectively reduced endothelial SP1 expression. SP1 is an important transcription factor that regulates cell functions including differentiation, proliferation and apoptosis. Accumulating evidence suggests that interactions between SP1 and NF‐κB P65 play key regulatory roles involved a variety of pathological processes. For example, silencing of SP1 protected H9C2 cells against LPS‐induced injury through binding to the promoter of CXCR4 and suppressing the NF‐κB P65 signalling pathway.^43^ In mature VSMCs, increased expression of NF‐κB P65 can reverse activation of the TRAIL promoter and promote VSMC proliferation after reduction of SP1.^31^, ^44^ Similarly, Lee et al. found that regulation of NF‐κB P65 and SP1 expression may be important targets for the prevention and treatment of atherosclerotic vascular disease.^28^ Therefore, we guessed that macrophage‐derived miR‐4532 sponged SP1 and leads to HUVEC injury through acting on NF‐κB P65, then we tested the relevance between SP1 and NF‐κB P65. Surprisingly, our data revealed that SP1 expression was prominently decreased following NF‐κB P65 paradoxically activated in ox‐LDL treated group compared with the mock group by Western blot. Further knockdown or overexpression of SP1 abolished the activation of NF‐κB P65 induced by exosomal miR‐4532 derived from ox‐LDL treated macrophages, following no change in ICAM‐1 and VCAM‐1 expression in HUVECs. These results indicated that exosomal miR‐4532 activated NF‐κB P65 and endothelial function relying on SP1. Consistently with our research data, Xue H et.al reported that lncRNA NKILA inhibited proliferation and promoted apoptosis of chondrocytes via miR‐145 targeting SP1 and activating NF‐κB P65.^45^ In conclusion, SP1, as an important transcription factor, might play a key role of macrophage‐derived exosomal miR‐4532 in HUVECs, while the detailed mechanism of how SP1 modulate NF‐κB P65 need further research to explore.

Just as our schematic diagram showed that ox‐LDL is taken up by macrophages, induces macrophages to become foam cells and release exosomes that contain high levels of miR‐4532, which are taken up by endothelial cells and inhibit SP1 expression, while activating NF‐κB P65 signalling, to promote ICAM‐1, VCAM‐1 expression, leading to endothelial dysfunction, and forming a vicious cycle. However, our study has several limitations. We focused primarily on the effect of macrophages on endothelial cells, and further exploration to include smooth muscle cells, which are also involved in atherosclerosis, is required. In addition, vivo study on animal model was lacked for the scarcity of mice miR‐4532 sequence, so our conclusions will be further supported by future animal experiments.

## 5 CONCLUSION

Collectively, our findings demonstrate that miR‐4532 in exosomes secreted by macrophages can be taken up by vascular endothelial cells, where it regulates SP1 expression and activates the NF‐κB P65, which promotes atherosclerosis. These findings may provide a new perspective for the development of treatment for atherosclerosis.

## AUTHOR CONTRIBUTIONS

**Guohai Su:** Project administration (equal); writing – review and editing (equal). **Peng Liu:** Methodology (equal); writing – original draft (equal). **Shuya Wang:** Investigation (equal); project administration (equal); supervision (equal). **Guangxin Wang:** Conceptualization (equal); supervision (equal). **Mingming Zhao:** Formal analysis (equal). **Fengli Du:** Formal analysis (equal). **Kaiyuan Li:** Methodology (equal). **Lei Wang:** Formal analysis (equal). **Yang Yang:** Supervision (equal). **Huihui Wu:** Supervision (equal). **Jiamin Chen:** Supervision (equal).

## CONFLICT OF INTEREST

The authors declared no conflicts of interest.

## Supporting information


Figure S1

## Data Availability

The data from public database supporting the findings of this study are available in the methods of this article. All raw data of this study are available on request from the corresponding author.
