# Supplementary material for: Macrophage‐derived exosomal miR‐4532 promotes endothelial cells injury by targeting SP1 and NF‐κB P65 signalling activation
Source: J Cell Mol Med. 2022 Sep 7;26(20):5165–80. doi: 10.1111/jcmm.17541 (PMC9575109; doi:10.1111/jcmm.17541)
Supplement: Supplementary file 1 — Figure S1 [file JCMM-26-5165-s001.docx]

**Supplementary Figure**

**
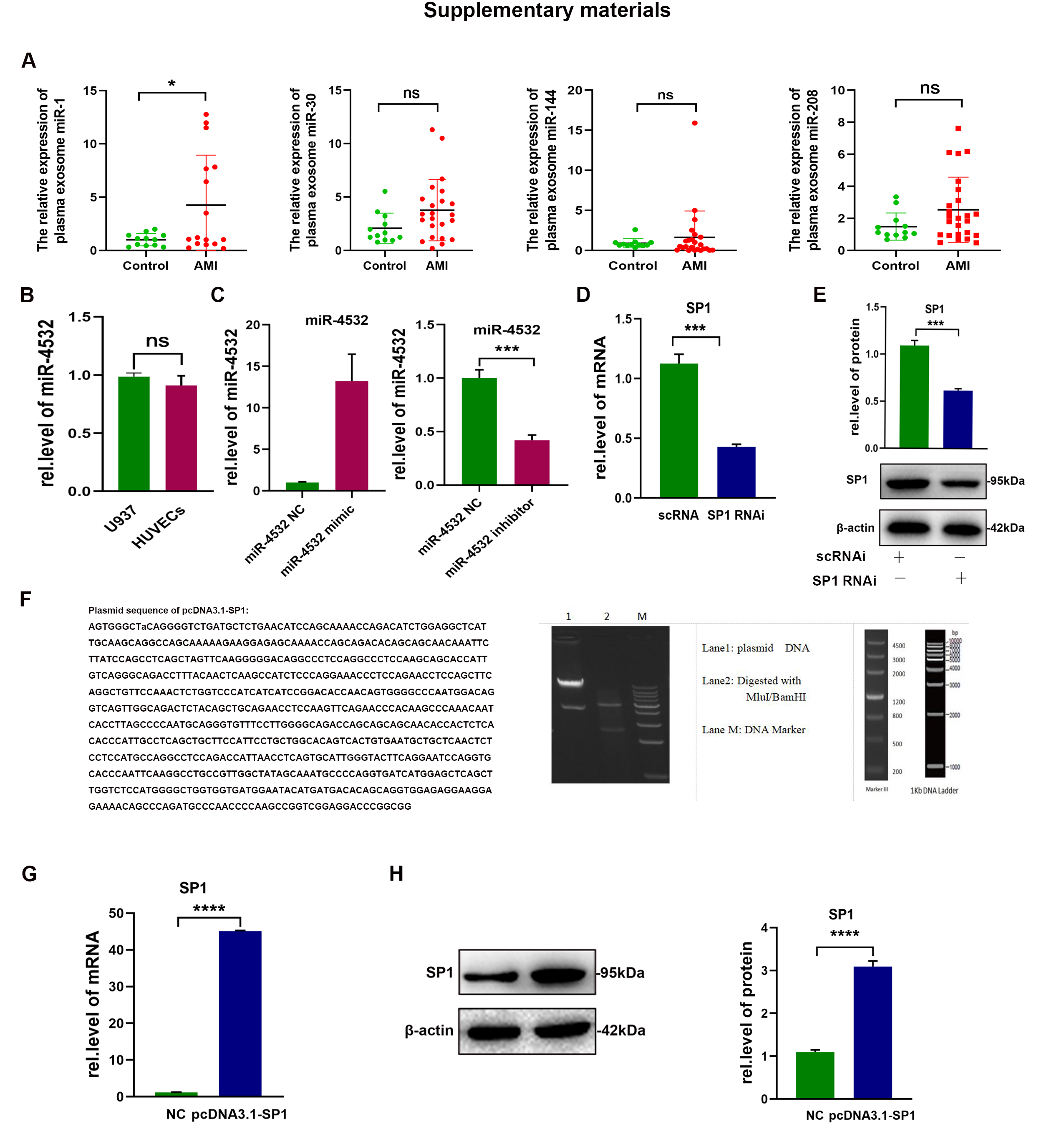
**

**Figure legend**

**(A)** There were no significant difference in the four miRNAs. The relative expression of miR-1-3p, miR-30-5p, miR-144-3p, and miR-203-3p in 20 patients with AMI and 12 controls were detected by qRT-PCR, which showed no significant difference. **(B)** The relative basic expression of miR-4532 had no difference between HUVECs and U937 cells. **(C)** Transfection efficiency of miR-4532 mimic or inhibitor in U937 cells were tested by qRT-PCR. **(D,E)** Knockdown of SP1 using specific RNAi decreased its relative expression in both mRNA and protein in HUVECs cells. **(F)** Plasmid sequence of pcDNA3.1-SP1 and the validation image were listed. **(G,H)** Relative intracellular expression of SP1 in both mRNA and protein level were dramatically elevated after plasmid transfection.* p < 0.05, ***p < 0.001, and ****p < 0.0001.
